# Body part categorical matching in chimpanzees (*Pan troglodytes*)

**DOI:** 10.1038/s41598-024-66829-w

**Published:** 2024-07-10

**Authors:** Jie Gao, Ikuma Adachi

**Affiliations:** 1 Hakubi Center and Wildlife Research Center, Kyoto University, 2-24 Tanaka-Sekiden-Cho, Sakyo, Kyoto 606-8203 Japan; 2 Center for the Evolutionary Origins of Human Behavior, Kyoto University, Inuyama, Japan

**Keywords:** Body perception, Body anatomy, Body image, Biological knowledge, Chimpanzees, Comparative cognition, Evolution, Psychology, Zoology

## Abstract

Humans categorize body parts, reflecting our knowledge about bodies, and this could be useful in higher-level activities involving bodies. We tested whether humans’ closest living relatives—chimpanzees—have the same ability using touchscreen tasks, focusing on the major parts: heads, torsos, arms, and legs. Six chimpanzees were trained to perform a body part matching-to-sample task using sets of pictures of chimpanzee bodies, where in each trial, the sample and choice pictures were the same. Five passed the training and received the test sessions, where three trial types were mixed: trained same-individual picture pairs; novel same-individual picture pairs; and novel different-individual picture pairs. All participants performed better than the chance level in all conditions and for all body parts. Further analyses showed differences in performance when the samples were different body parts. For example, the results revealed better performances for heads and torsos than arms and legs in “novel different-individual pairs”. The study showed that chimpanzees can visually match and categorize body parts in this experiment setting, even across different chimpanzees’ bodies, suggesting potential biological understanding. Different performances for body parts suggested a deviated categorization from humans. We hope this study will inspire future research on the evolution of body perception.

## Introduction

Bodies are vital for animals, not only in a literal sense, but also because they convey many social cues. Many animal species use their bodies to demonstrate various behaviors and intentions, and during interactions, other individuals’ bodies often convey signals that are crucial to daily life. Bodily orientations and postures may indicate specific directions of movement and other behaviors, offering a basis for others to make key decisions in subsequent interactions. Researchers have identified a gesture repertoire in chimpanzees that is used for daily social communications^1–3^. This repertoire encompasses approximately 60 gesture types that involve multiple anatomical parts, with each gesture conveying specific social meanings. Some gestures are body-part specific. For example, “foot present” mean “climb on me”, while “arm raise” means “acquire object” and “hand fling” means “move away”^2^. Chimpanzees also use their bodies to manipulate tools—for example, in ant dipping, termite fishing, and nut cracking—and evidence indicates that social learning is an essential means of disseminating tool-using culture^4–8^. Social learning requires chimpanzees to pay attention to tools, as well as the body parts that manipulate them. Chimpanzees’ active use of their bodies in daily life suggests that they have a certain level of knowledge about their anatomies. For example, to understand different gestures, they may need to be able to distinguish and categorize various anatomical parts. This anatomical knowledge may also facilitate their understanding of others’ behavior and intentions, communications using bodily gestures, and social learning.

Anatomical knowledge also contributes to body image cognition, a fundamental element of species and individual recognition. Chimpanzees show significantly better performance in recognizing conspecifics’ bodies when the bodies are upright than when they are inverted^9^, indicating a specialized—and likely more efficient—way of detecting and processing information derived from bodies. When the body part arrangement was disrupted, this inversion effect dissipated^10^, suggesting that specialized body processing is based on correct body part arrangement. In an eye-tracking study, it was found that chimpanzees looked longer at atypical body parts compared to the body parts in typical body pictures^11^, which also suggests that they can detect unfamiliar elements on conspecifics’ bodies. However, few studies have examined chimpanzees’ representations and categorization of body parts in depth.

We are particularly interested in body part categorization, which is a major factor influencing how bodies are perceived that relates to the perception and understanding of body parts. Clear and consistent body part categorization may allow animals to better understand and use various anatomical cues. In a food request study, researchers examined if chimpanzees could use the cue of visibility of different body parts of humans to get food, but the results suggested a failure in reasoning about the use of human limbs^12^. In a more direct study in facial part matching in chimpanzees, it was found that although the ability to match facial parts across two faces seemed unreliable, they did succeed in several cases, including matching ears of chimpanzees and humans, despite that the ear locations in the two species differ^13^. This suggests possible abilities for chimpanzees to represent different body parts. However, chimpanzees’ body part categorization has yet to be assessed directly. Studies have shown that human children can typically begin to name different body parts from approximately 2 years old^14–18^. Chimpanzees are humans’ closest living relatives, and like humans, they engage in gestural communication, use tools, and engage in many social interactions actively using their body parts; unlike humans, however, they may not have the language to distinguish different body parts. Therefore, investigation of chimpanzees’ body part categorization may help us understand how they perceive body image without the use of language while simultaneously enriching our understanding of how knowledge about bodies has evolved.

Chimpanzees’ bodies are similar to those of humans with respect to shape and structure: the head on top, the torso in the center, and four limbs. Actually, during the evolution, this body form is largely reserved in many species. Although they use quadrupedal postures more frequently, they manipulate objects mainly using their arms and hands, just as humans do^4,7^. They also have similar visual abilities to humans and a similar specialized way of visually processing body images^9,19^. Therefore, they may also have a similar approach to categorization. While animal species that use bodily cues and gestures might all have a certain level of body part representation, in this study, we examined chimpanzees first. Based on similarities in body forms and visual perception in chimpanzees and humans, we adopt a top-down approach to examine chimpanzees’ body part categorization. We designed the experiment based on humans’ general body part categorization, which also describes the universal forms of many species, especially mammals (four major parts: head, torso, arms, and legs) to assess chimpanzees and infer their body part categorization ability based on their test performance, with the aim of determining whether chimpanzees can match body parts under this categorization framework. Performance differences with respect to the categorization of each body part may allow us to infer whether and how their categorization differs from that of humans.

**Figure 1:**
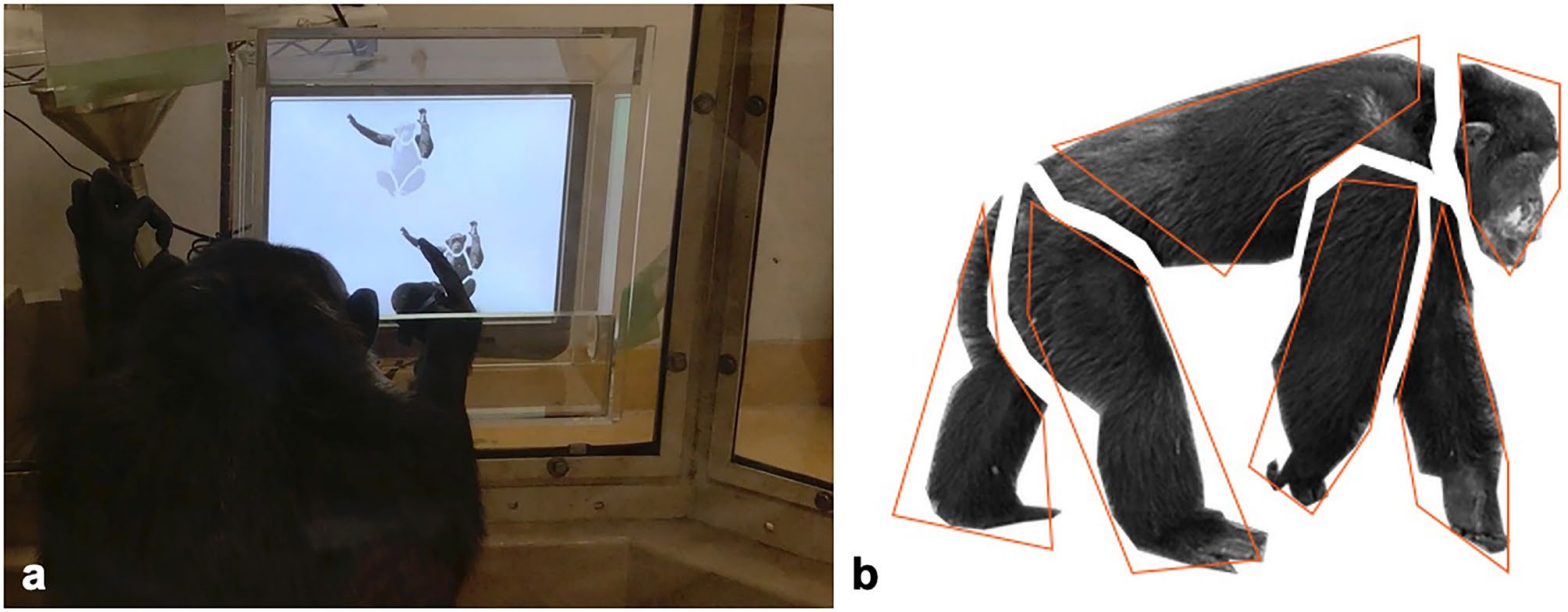
The setting of the experiment (**a**) and an example stimulus (**b**). (**a**) Cleo sat in front of a touchscreen and engaged in the task. (**b**) The red-lined pentagons show the designated areas for each body part. These lines are not shown during the experiment.

In this study, we tested chimpanzees using a body part-matching task with touchscreen devices, with the overall aim of understanding their approach to body part categorization relative to humans’ general approach. An early study in macaques has confirmed that the animals do understand that the faces shown on slides are representations of real animal individuals^20^. A study in chimpanzees using similar settings with the current study also found that they could match vocalizations with individuals’ face representation, suggesting that chimpanzees understand the true meaning of 2D representations on a screen^21^. Therefore, we use images on the touchscreens to represent chimpanzee bodies and believe this is a valid design to study their categorization of body parts. We used images of chimpanzee bodies as stimuli and defined four major parts: head, torso, arms, and legs. The experiments trained chimpanzees to match the body parts across sample pictures and then tested them using novel pictures (Fig. 1). Based on their accuracy, their choices under each condition, and differences across conditions, we can infer how chimpanzees’ body part categorization differs from humans’ categorization of heads, torsos, arms, and legs.

Six chimpanzees were trained first in the pre-training stage to learn touching the target body parts (indicated by a flashing visual effect) on chimpanzee body images, then in the training stage to learn the body part matching-to-sample paradigm. In this paradigm, for each trial, one chimpanzee body image with a body part flashing (the head, torso, arms, or legs) appeared, and after they touched the target flashing part, another body image appeared and they needed to touch the same part on the new body image to get food reward, otherwise there was no food reward. The training stage had three phases using different sets of stimuli, while in each trial, the sample and choice bodies were the same images. Five chimpanzees passed the criteria for training and proceeded to the final testing stage. In the testing stage, three different types of trials were mixed: “baseline” trials, where the stimuli were those used in the training stage, and the same images appeared in each trial; “test_same” trials, where the stimuli were novel, and the same images appeared in each trial; and “test_different” trials, where the stimuli were novel, and different images were used within each trial. The purpose of the “test_same” trials was to provide a condition to examine the generalization of the categorization but at the same time keep the pattern the same as training and baseline trials, where both stimuli were the same within one trial. The purpose of the “test_different” trials was to further examine how they categorize body parts when the two bodies were different. If the participants show different performances in these three conditions, it may suggest an effect from stimulus familiarity and similarity on discrimination and categorization. If their performances are better than the chance level, then it means despite the basic effects from stimulus familiarity and similarity, they could still complete the task to correctly match body parts. We examined their accuracies of choices under different conditions and compared the accuracies and response times across the body parts.

## Results

### Number of training sessions

Table 1 details the numbers of pre-training and training sessions for each individual. All chimpanzees except Ayumu completed 9.3 ± 2.5 training sessions on average.

**Table 1:** The session numbers of the pre-training and training stages for each individual.

Participant	Pretraining	Training, Phase 1	Training, Phase 2	Training, Phase 3	Training, average
Ai	15	13	4	4	7
Ayumu	15	21	45^a^	n.a	n.a
Chloe	35	12	6	5	7.7
Cleo	15	2	46	11	16.7
Pal	15	7	4	8	6.3
Pendesa	15	9	10	8	9

^a^Ayumu was unable to complete the training because he did not come to the lab often.

### Accuracy of different conditions and different body parts

Table 2 shows the accuracy for each individual in the “baseline” condition and the “test_same” condition, the accuracy for each individual and body part in the “test_different” condition, the accuracy for each individual when they encountered the stimuli for the first time in the “test_different” condition, and the binomial test results (compared with the chance level, 25%). All chimpanzees performed significantly better than the chance level.

**Table 2:** Accuracy (%) for each individual in the “baseline” condition and the “test_same” condition, for each individual and body part in the “test_different” condition, and for each individual when they first encountered each stimulus pair in the “test_different” condition, as well as the binomial test results.

Individual	Baseline, All		Test_Same, All		Test_Different, All		Test_Different, Head^a^		Test_Different, Torso^a^		Test_Different, Arm^a^		Test_Different, Leg^a^		Test_Different, first trials	
	Accuracy	*P*	Accuracy	*P*	Accuracy	*P*	Accuracy	*P*	Accuracy	*P*	Accuracy	*P*	Accuracy	*P*	Accuracy	*P*
Ai	86	< 0.001	88	< 0.001	71	< 0.001	98	< 0.001	85	< 0.001	62	< 0.001	40	.004	65	< 0.001
Chloe	93	< 0.001	93	< 0.001	76	< 0.001	97	< 0.001	72	< 0.001	75	< 0.001	60	< 0.001	70	< 0.001
Cleo	92	< 0.001	92	< 0.001	79	< 0.001	95	< 0.001	88	< 0.001	70	< 0.001	63	< 0.001	80	< 0.001
Pal	94	< 0.001	91	< 0.001	70	< 0.001	82	< 0.001	92	< 0.001	63	< 0.001	43	< 0.001	65	< 0.001
Pendesa	86	< 0.001	88	< 0.001	71	< 0.001	97	< 0.001	77	< 0.001	72	< 0.001	55	< 0.001	70	< 0.001

^a^Refers to the sample part.

### Testing stage, accuracy data

**Figure 2:**
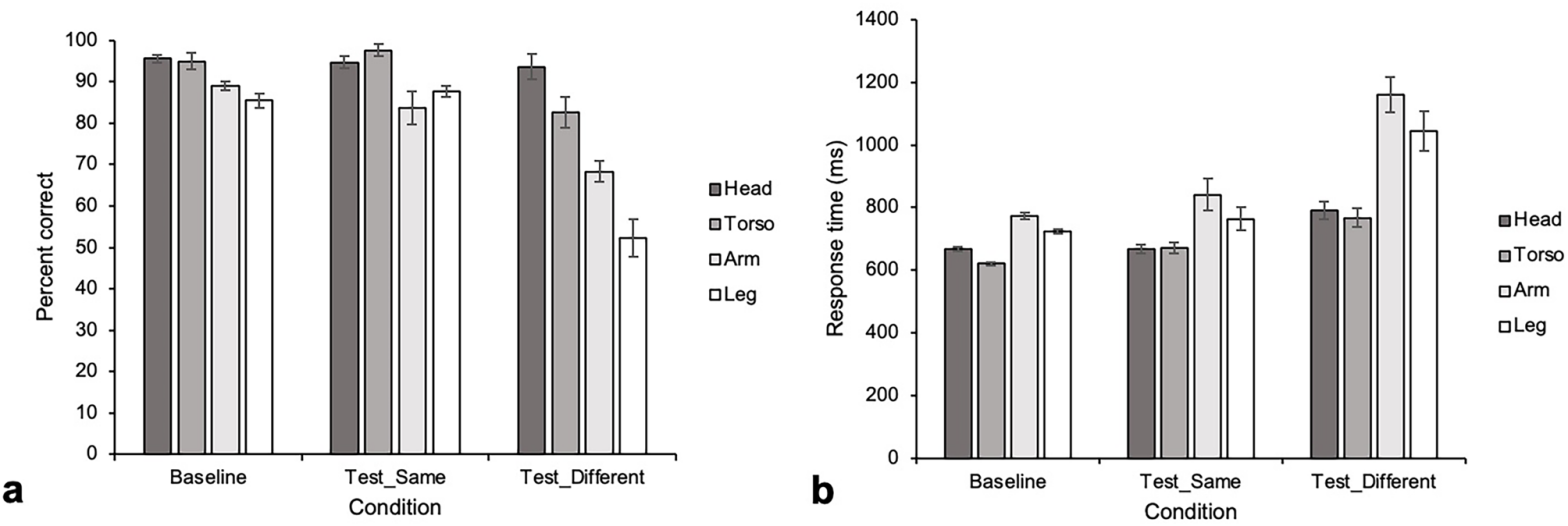
The mean accuracy and response time of each condition for each body part. (**a**) Accuracy data. Error bar: SEM. (**b**) Response time data. Error bar: SEM.

For the testing stage, the average accuracies were as follows (Fig. 2a): “baseline” condition: 95.6 ± 0.9% (head), 95.0 ± 1.9% (torso), 89.0 ± 1.1% (arm), and 85.4 ± 1.7% (leg); “test_same” condition: 94.7 ± 1.4% (head), 97.7 ± 1.5% (torso), 83.7 ± 4.1% (arm), and 87.7 ± 1.2% (leg); and “test_different” condition: 93.7 ± 3.0% (head), 82.7 ± 3.7% (torso), 68.3 ± 2.5% (arm), and 52.3 ± 4.6% (leg). The full model with fixed effects of condition, body part and their interaction was significantly different from the null model (χ^2^ [11, *N* = 6] = 495.2, *p* < 0.001). For the full model, analysis of variance based on mixed logistic regression indicated significant effects on accuracy of the interaction of condition and body part (χ^2^ [6, *N* = 6] = 39.5, *p* < 0.001), condition (χ^2^ [2, *N* = 6] = 28.6, *p* < 0.001), and body part (χ^2^ [3, *N* = 6] = 231.7, *p* < 0.001). The results of simultaneous pairwise comparisons based on either condition or body part are shown in Table 3. Particularly, in the “test_different” condition, accuracy differed significantly among body parts in the following order (from high to low): head, torso, arm, leg. The variance values of the random effects for session number, trial number, sample pictures, picture location, and participant were 0.015, < 0.001, 0.20, 0.052, and 0.065, respectively; the standard deviation (*SD*) values were 0.122, < 0.001, 0.452, 0.227, and 0.256, respectively.

**Table 3:** Results of post hoc pairwise comparison of accuracy in the testing stage based on the interaction between condition and sample body part.

Sample body part	Condition	Contrast	Estimate	*SE*	*Z* ratio	*P* value
Arm	–	Baseline—test_different	1.352	0.253	5.337	< 0.001
Arm	–	Baseline—test_same	0.476	0.305	1.561	0.977
Arm	–	Test_different—test_same	− 0.875	0.322	− 2.716	0.181
Head	–	Baseline—test_different	0.318	0.332	0.956	1.000
Head	–	Baseline—test_same	0.169	0.373	0.453	1.000
Head	–	Test_different—test_same	− 0.149	0.432	− 0.344	1.000
Leg	–	Baseline—test_different	1.738	0.247	7.030	< 0.001
Leg	–	Baseline—test_same	− 0.221	0.314	− 0.705	1.000
Leg	–	Test_different—test_same	− 1.959	0.329	− 5.961	< 0.001
Torso	–	Baseline—test_different	1.345	0.276	4.865	< 0.001
Torso	–	Baseline—test_same	− 0.816	0.466	− 1.752	0.917
Torso	–	Test_different—test_same	− 2.161	0.482	− 4.486	< 0.001
–	Baseline	Arm—head	− 1.008	0.113	− 8.936	< 0.001
–	Baseline	Arm—leg	0.331	0.083	3.988	0.002
–	Baseline	Arm—torso	− 0.875	0.108	− 8.102	< 0.001
–	Baseline	Head—leg	1.339	0.109	12.265	< 0.001
–	Baseline	Head—torso	0.132	0.129	1.025	1.000
–	Baseline	Leg—torso	− 1.206	0.104	− 11.570	< 0.001
–	Test_different	Arm—head	− 2.041	0.273	− 7.481	< 0.001
–	Test_different	Arm—leg	0.717	0.178	4.033	0.0017
–	Test_different	Arm—torso	− 0.882	0.204	− 4.325	< 0.001
–	Test_different	Head—leg	2.759	0.270	10.205	< 0.001
–	Test_different	Head—torso	1.159	0.286	4.051	0.0015
–	Test_different	Leg—torso	− 1.599	0.200	− 7.997	< 0.001
–	Test_same	Arm—head	− 1.315	0.302	− 4.355	< 0.001
–	Test_same	Arm—leg	− 0.367	0.237	− 1.545	0.980
–	Test_same	Arm—torso	− 2.168	0.412	− 5.262	< 0.001
–	Test_same	Head—leg	0.948	0.312	3.040	0.069
–	Test_same	Head—torso	− 0.853	0.459	− 1.860	0.858
–	Test_same	Leg—torso	− 1.801	0.419	− 4.295	< 0.001

### Testing stage, response time data

The response times in the testing phase were as follows (Fig. 2b): “baseline” condition: 668 ± 6.7 ms (head), 621 ± 6.1 ms (torso), 774 ± 10.4 (arm), and 725 ± 7.4 ms (leg); “test_same” condition: 667 ± 13.6 ms (head), 672 ± 17.4 ms (torso), 841 ± 50.4 ms (arm), and 764 ± 36.6 ms (leg); and “test_different” condition: 791 ± 28.5 ms (head), 768 ± 30.9 ms (torso), 1,160 ± 55.4 ms (arm), and 1,044 ± 64.6 ms (leg). The full model with fixed effects of condition, body part, and their interaction differed significantly from the null model (χ^2^ [11, *N* = 6] = 986.9, *p* < 0.001). For the full model, analysis of variance based on mixed logistic regression indicated significant effects on accuracy of the interaction between condition and body part (χ^2^ [6, *N* = 6] = 17.4, *p* = 0.0079), condition (χ^2^ [2, *N* = 6] = 49.6, *p* < 0.001), and body part (χ^2^ [3, *N* = 6] = 257.0, *p* < 0.001). Table 4 presents the results of simultaneous pairwise comparisons based on condition or body part. Particularly, in the “test_different” condition, the response times of the trials in which the samples were arms or legs were significantly longer than those of the trials in which the samples were heads or torsos. The variance values of the random effects of session number, trial number, sample pictures, picture location, and participant were < 0.001, < 0.001, < 0.001, < 0.001, and 0.19, respectively; and their *SD* values were < 0.001, < 0.001, < 0.001, 0.009, and 0.436, respectively.

**Table 4:** Results of post hoc pairwise comparison of response time in the testing stage based on the interaction between condition and sample body part.

Sample body part	Condition	Contrast	Estimate	*SE*	*Z* ratio	*P* value
Arm	–	Baseline—test_different	4.20e − 04	6.02e − 05	6.97	< 0.001
Arm	–	Baseline—test_same	1.02e − 04	7.26e − 05	1.41	0.0011
Arm	–	Test_different—test_same	− 3.18e − 04	7.73e − 05	− 4.11	0.0011
Head	–	Baseline—test_different	2.33e − 04	6.46e − 05	3.61	0.0080
Head	–	Baseline—test_same	1.03e − 06	7.69e − 05	0.013	1
Head	–	Test_different—test_same	− 2.32e − 04	8.41e − 05	− 2.76	0.11
Leg	–	Baseline—test_different	4.02e − 04	6.47e − 05	6.21	< 0.001
Leg	–	Baseline—test_same	1.14e − 04	7.36e − 05	1.55	0.80
Leg	–	Test_different—test_same	− 2.88e − 04	8.12e − 05	− 3.54	0.010
Torso	–	Baseline—test_different	3.17e − 04	6.73e − 05	4.71	< 0.001
Torso	–	Baseline—test_same	1.30e − 04	7.66e − 05	1.69	0.71
Torso	–	Test_different—test_same	− 1.87e − 04	8.54e − 05	− 2.19	0.37
–	Baseline	Arm—head	− 2.08e − 04	2.04e − 05	− 10.16	< 0.001
–	Baseline	Arm—leg	− 8.65e − 05	2.01e − 05	− 4.30	< 0.001
–	Baseline	Arm—torso	− 3.19e − 04	2.13e − 05	14.94	< 0.001
–	Baseline	Head—leg	1.21e − 04	2.12e − 05	5.71	< 0.001
–	Baseline	Head—torso	1.11e − 04	2.24e − 05	− 4.95	< 0.001
–	Baseline	Leg—torso	− 2.32e − 04	2.21e − 05	− 10.49	< 0.001
–	Test_different	Arm—head	− 3.94e − 04	4.99e − 05	− 7.90	< 0.001
–	Test_different	Arm—leg	− 1.05e − 04	4.96e − 05	− 2.11	0.43
–	Test_different	Arm—torso	− 4.22e − 04	5.30e − 05	− 7.95	< 0.001
–	Test_different	Head—leg	2.90e − 04	5.50e − 05	5.27	< 0.001
–	Test_different	Head—torso	− 2.75e − 05	5.76e − 05	− 0.48	1
–	Test_different	Leg—torso	− 3.17e − 04	5.78e − 05	− 5.49	< 0.001
–	Test_same	Arm—head	− 3.09e − 04	6.00e − 05	− 5.15	< 0.001
–	Test_same	Arm—leg	− 7.44e − 05	5.56e − 05	− 1.34	0.90
–	Test_same	Arm—torso	− 2.91e − 04	5.92e − 05	− 4.91	< 0.001
–	Test_same	Head—leg	2.34e − 04	6.09e − 05	3.85	0.0032
–	Test_same	Head—torso	1.79e − 05	6.40e − 05	0.28	1
–	Test_same	Leg—torso	− 2.16e − 04	6.01e − 05	− 3.60	0.0082

### Multi-dimensional scaling analysis, “test_different” condition of the testing stage

**Figure 3:**
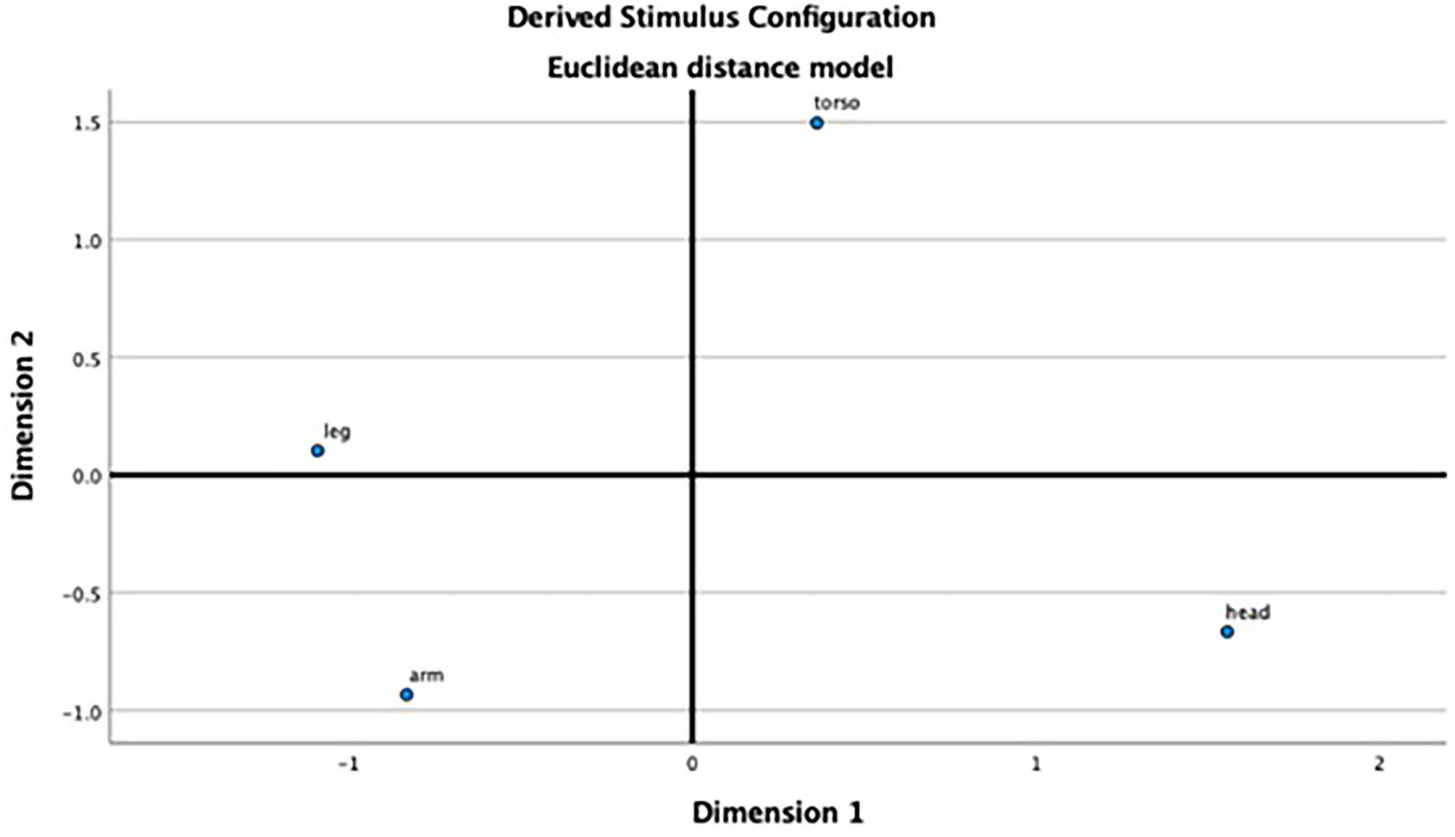
Results of the multi-dimensional scaling analysis. Stress = 0.175, squared correlation = 0.902.

Based on all participants’ choices for each body part in the “test_different” condition (Table 5), we performed a multi-dimensional scaling analysis (Fig. 3) to determine how well they discriminated body parts. The coordinates of the four body parts were as follows: head [1.56, − 0.67], torso [0.36, 1.50], arm [− 0.83, − 0.93], and leg [− 1.09, 0.10]. The stress value was 0.175, and *r*^*2*^ was 0.902.

**Table 5:** Choices made for each sample by all individuals in the “test_different” condition.

Sample	Choice				
	Head	Torso	Arm	Leg	Sum
Head	281	7	3	9	300
Torso	13	248	23	16	300
Arm	8	16	205	71	300
Leg	9	52	82	157	300

## Discussion

In this study, we tested six chimpanzees (five of whom passed the training phases and proceeded to the final testing tasks). We used a body part matching-to-sample task to examine how they matched four major body parts—head, torso, arms, and legs—across the same or different chimpanzee body images on touchscreens to assess their body part categorization ability. We first trained them to match the same body parts across two identical chimpanzee body images using a series of samples. After they passed the training phase, we mixed the trained pairs (“baseline”) and novel pairs in the testing phase. The novel pairs included both same-picture pairs (“test_same”) and different-picture pairs (“test_different”). We examined the chimpanzees’ performances in the testing stage. Accuracy was significantly higher than the chance level for each participant in each condition; for each body part in the “test_different” condition; and for each individual when they first encountered the novel pairs in the “test_different” condition. The accuracy for all chimpanzees differed significantly according to condition, body part, and their interaction. The post hoc pairwise comparison showed significant differences in accuracy among the four body parts in the “test_different” condition (from highest to lowest: head, torso, arms, and legs). The response time for all chimpanzees also differed significantly according to condition, body part, and their interaction, and the post hoc pairwise comparison showed that, for the head and torso, the response times were significantly shorter than for arms and legs. We also did a multi-dimensional scaling analysis to determine how well the chimpanzees discriminate the four body parts. The distance between arms and legs was relatively short, suggesting that the chimpanzees were most confused about the arms and legs. The results clearly suggest that the chimpanzees were able to match body parts even across different chimpanzee body images in a setting of touchscreen tasks. Some differences emerged in their performance among the four body parts, indicating that their ability to discriminate differed among the body parts; thus, their body part categorization may deviate from the clear “head, torso, arms, and legs” pattern seen in humans.

The results from the binomial tests, and the fact that the accuracy values were relatively high, suggest that the participants were able to match body parts without difficulty. However, further analysis revealed lower accuracies in the “test_different” condition, and lower accuracies and longer response times for certain body parts. Nonetheless, performance was better than the chance level in the “test_different” condition for all body parts, and the similar results for the first encounters with all novel pairs indicate clear understanding that the four body parts—head, torso, arms, and legs—are different; only the extent of discrimination differed, possibly indicating a different approach to grouping body parts than “head, torso, arms, and legs”. This is the first evidence from cognitive tests of chimpanzees’ body part recognition ability. The ability to discriminate body parts is essential because behaviors are produced using different body parts. For example, different combinations of body parts form various bodily gestures for social communication^1–3^; it also might be important to discriminate body parts involved in tool use, via social learning, to obtain food and resources^4–8^. We still know little about the potential mechanisms by which chimpanzees’ understanding of body parts facilitates learning and understanding of behaviors and intentions. Future studies may provide greater insight into body part recognition and thereby further elucidate the connection between chimpanzees’ body knowledge and behavior reading.

The results also revealed interesting differences in the chimpanzees’ performance among conditions and body parts. Regarding conditions, in many pairwise comparisons (Tables 3 and 4), performance in the “test_different” condition was always significantly worse than in the “baseline” condition (accuracy: arm, leg, torso; response time: all four body parts). In some pairs, there were significant differences between the “test_same” condition and “test_different” condition (accuracy: leg and torso; response time: arm and leg), and there was also a difference between the “baseline” and “test_same” conditions, albeit only for one pair (response time: arm). Generally, performance was the worst in the “test_different” condition, which was the only condition that used different individuals in the same pair in the matching task. The pairs in the “test_same” condition included the same pictures within each pair, so it was still easy despite the fact that all the pictures were novel to the participants. The fact that they performed well in these novel-but-same picture pairs validates the training. When the pictures were different, the cognitive load became heavier, and the chimpanzees made more mistakes or took longer to complete the task. In the “test_different” condition, accuracy differed significantly among the four body parts, for all combinations (from highest to lowest: head, torso, arms, and legs). The response times showed no significant differences when we compared arms with legs and head with torso, although head and torso had shorter response times than arms and legs. A similar pattern emerged in other conditions: the response times either all differed from each other, or those of the head and torso were similarly superior to those of arms and legs. The difference in matching performance among body parts could have been caused by both low-level features and high-level cognitive mechanisms. Low-level features, such as sizes, shapes and locations, may have informed the chimpanzees’ judgments, and higher-level knowledge, such as their daily use of different body parts, may also have influenced their choices in the task. The head and torso may be easier to discriminate because there is only one of each, whereas there are four limbs. The face, which conveys multiple social cues, including identity and emotions^22,23^, is part of the head and is thus likely to be highly salient for chimpanzees. We also cannot simply rule out an influence of long-term experience with cognitive tasks, many of which require specific matching of faces^24–27^. Given that the torso is always at the body’s center and is larger than the other parts, chimpanzees may find it easier to match the torso across different images. Nevertheless, although the head is much smaller than the torso, the accuracy was significantly higher in the “test_different” condition, therefore, the low-level features are not determinative in their decisions.

The results of the multi-dimensional analyses (Fig. 3) suggest a relatively good representation since the stress value was low (0.175) and the *r*^*2*^ was high (0.902). The four body parts clearly distribute in different areas, showing a relatively clear discrimination among these four parts. At the same time, the shorter distance between “arms” and “legs” suggests that they were most confused about these two parts. The GLMM analyses indicated the same conclusion. Some low-level visual features, such as similarity of shapes, may have caused some difficulties. The locations and gestures may also have confused the participants—for example, it may be easier to discriminate between arms and legs when the two pictures are side by side rather than corner to corner, and when the chimpanzee in the picture is in a bipedal standing posture rather than a quadrupedal side view. Moreover, regarding chimpanzees’ greater experience with certain body parts, their typical quadrupedal postures may make them less capable of discriminating arms and legs than bipedal humans^4,7^. Like humans, chimpanzees still manipulate many objects using only their arms and hands^4,7^. However, many of their bodily gestures involve their legs and feet, while humans’ gestures typically do not^1–3^. Chimpanzees also climb a lot, using both their arms and legs, while humans discriminate more in terms of the use of their arms and legs in daily life. The closeness in function may have influenced performance in the body part-matching task.

One may argue whether the results from this study truly reflect chimpanzees’ ability to categorize body parts, other than merely completing an object discrimination and matching task. We agree that we need to be cautious when making this conclusion, but still, the design and chimpanzees’ performances are in favor of them being able to actually match body parts. First of all, although previous studies already suggested that chimpanzees in this experimental setting can understand the meaning of the 2D images^20,21^, the relatively small presentation size of bodies in this study might affect their association between the images to real-life objects. Also, the experiment design is at a visual level. Thus, the positive results may not transfer to other forms of testing, including pointing and matching body parts across real-life bodies (although this experiment may be difficult to conduct in chimpanzees) as the latter may involve more modules of cognitive abilities. Therefore, we must be cautious not to over-generalize the conclusion. On the other hand, this task is different from a simple object matching. The positive results in the “test_different” clearly show evidence for a body-part matching, because the two body images are completely different, sometimes even with the gestures being different from each other (see Supplementary materials). That changes the detailed shapes, absolute positions, and relative positions of the same body parts. Chimpanzees have a much better performance than the chance level in this condition, suggesting a categorization for the true objects, body parts, instead of simple shapes. Nonetheless, various future tests could be done to make this case more persuasive.

One limitation of this study is that we did not include other types of controls, which would have allowed us to determine whether the matching was domain-specific or domain-general. Researchers investigating children’s body knowledge have found many features characteristic of domain-specific capabilities, suggesting that, beyond the physical and psychological domains, children develop knowledge specific to the biological domain^28^. This suggests that a set of perceptual and cognitive features are in place to aid biological understanding. Regarding body part categorization in chimpanzees, future studies should further examine the underlying mechanisms, perhaps through comparison with their categorization of other objects at a similar level of discrimination. This would permit us to infer whether chimpanzees, like humans, also have a specific set of cognitive features devoted to understanding the biological world. If they use a domain-specific strategy similar to faces, then examining their performances using inverted stimuli will generate useful information, as recognition for both faces and bodies is affected by inversion^9,24^. Furthermore, testing their categorization of body parts across different species would also be informative in terms of their biological knowledge.

In this study, we used images of chimpanzees in different postures. For the training phase, we used nine chimpanzee images, three of which depicted them in bipedal standing postures, three in sitting postures, and three in quadrupedal standing postures. We also mixed these different postures in the images used in the testing phase. Because the number of images depicting each posture type was limited, it is difficult to determine precisely how posture affected the participants’ performance. Future studies should further investigate the impact of this aspect on the results. Testing body knowledge using rotated body images might also yield more information on how they use cues from bodies. Besides postures, testing with body images of individuals of different ages or sizes may further elucidate the representation and categorization of body parts. Body part proportions change with age and in different sizes. Examining this could help understand how body part perception changes with different variables.

Another potential future direction would be to compare chimpanzees with human adults and children using the same paradigm. Although we may think categorizing body parts as head, torso, arms, and legs is obvious, further testing using the same paradigm may not support this. For younger children and toddlers, body part categorizations may change as they learn to move bipedally. Comparison of chimpanzees with humans in similar tests will help elucidate precisely how the species differ and how those differences may come to exist.

In summary, we tested chimpanzees’ body part categorization in a body part matching-to-sample task based on humans’ body part categorization (head, torso, arms, and legs). We found that chimpanzees are able to match these body parts but performance differed among the parts, indicating a categorization approach that likely deviates from that employed by humans. Future studies should continue to probe this topic by examining the mechanisms underlying chimpanzees’ categorization (domain-specific or domain-general), examining the effects of body postures, and conducting comparative studies across species. We hope that this study will support future explorations of animals’ body knowledge, the link between body knowledge and the daily use of bodies, and how we evolved to understand the biological world.

## Methods

### Participants

Six adult chimpanzees at the Center for the Evolutionary Origins of Human Behavior (EHUB) of Kyoto University participated in the experiment (Table 6). They lived in two social groups (12 individuals in total). Their living areas included an outdoor compound with attached indoor compounds, enriched with high climbing frames and many plants^29^. The chimpanzees participated in cognitive tests on a daily basis. They had full access to food and water during the study. All individuals were born in captivity except for Ai, who was brought to EHUB from the wild when she was about 1 year old (details are available from the Great Ape Information Network; see Table 6). The chimpanzees all had extensive experience of using touchscreens in many task paradigms, including discrimination tasks, visual search tasks, and classic matching-to-sample tasks, but not in the exact type of task used in this study (e.g.,^9,27,30^). The chimpanzees’ daily care and use adhered to the 2010 Guidelines for the Care and Use of Laboratory Primates of EHUB. The research proposal was approved by the Animal Welfare and Animal Care Committee of EHUB and the Animal Research Committee of Kyoto University (#2020-118, #2021-139, #2022-096). All procedures adhered to the Japanese Act on the Welfare and Management of Animals.

**Table 6:** General characteristics of the six chimpanzees.

Name	GAIN ID number^a^	Sex	Age (when the study started)	Kinship
Ai	0434	Female	44	Ayumu’s mother
Ayumu	0608	Male	20	Ai’s son; Pal’s paternal half sibling
Chloe	0441	Female	39	Cleo’s mother
Cleo	0609	Female	20	Chloe’s daughter
Pal	0611	Female	20	Ayumu’s paternal half sibling
Pendesa	0095	Female	43	–

^a^Identification number for each chimpanzee listed in the database of the Great Ape Information Network (GAIN; https://shigen.nig.ac.jp/gain/).

### Apparatus

The participants came to the lab booth and performed the tasks on a touchscreen computer with a 17-in. LCD monitor (I-O Data LCD-AD172F2-T; 1280 × 1024 pixels [px]; Fig. 1a). The viewing distance was approximately 40 cm. The participants could move freely during the experiment, but they usually sat in a natural and relaxed position while performing the task. A feeder automatically delivered a piece of apple or a raisin to the participants via a tube when they made a correct choice. The experiment was controlled by a computer using a program written with Microsoft® Visual Basic® 2010 software (Microsoft Corp., Redmond, WA, USA).

### Stimuli

**Figure 4:**
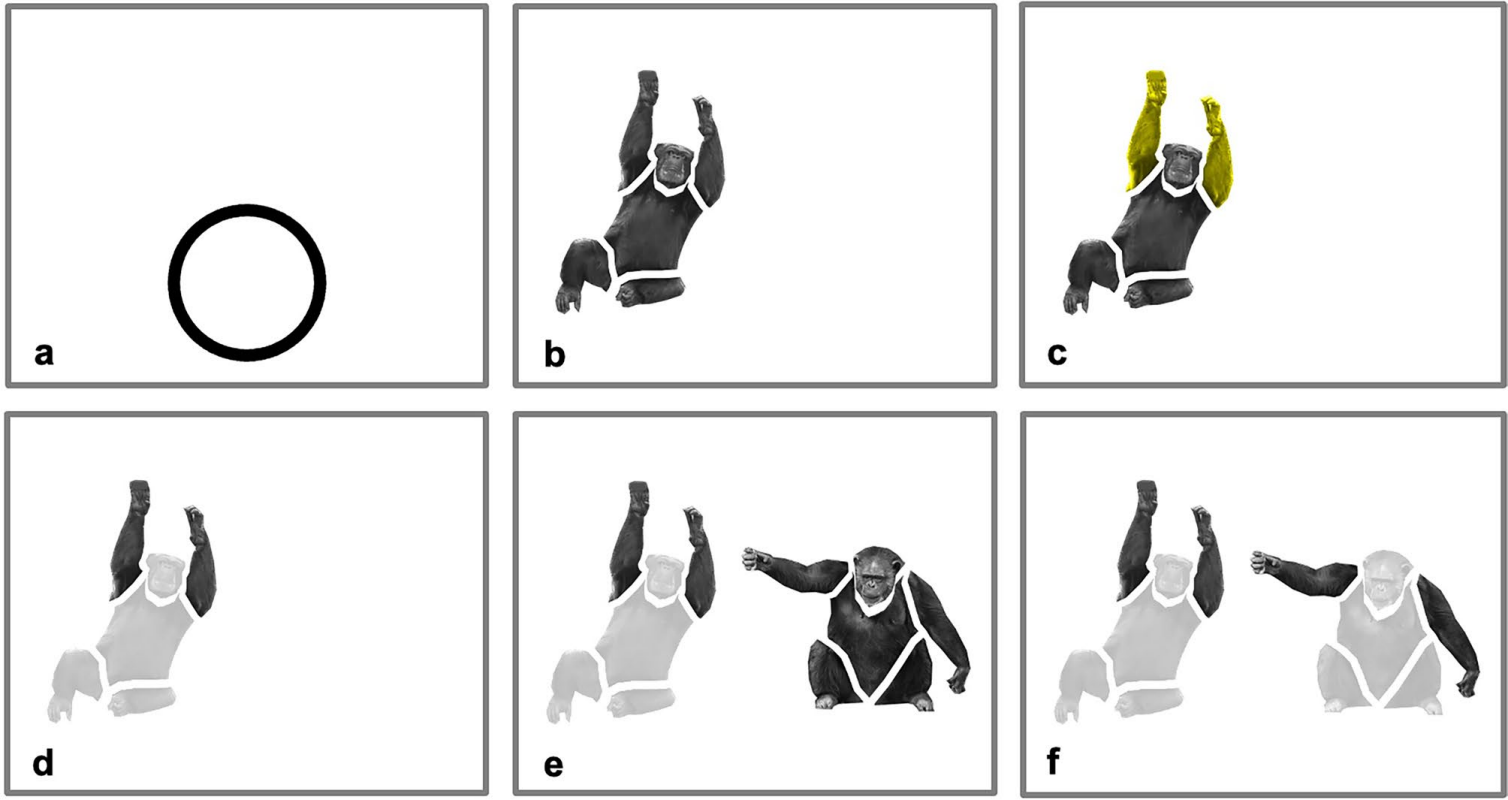
An example trial in the final testing stage. (**a**) At the beginning of each trial, a starting key appeared in the bottom center of the screen. (**b**) After chimpanzees touched the starting key, an image of a chimpanzee body with all body parts at a slight distance from each other appeared in one location on the screen. (**c**) The same picture with the targeted body parts highlighted in a yellow hue quickly took turns to appear with the image in (**b**), create a flashing visual effect for three rounds. (**d**) Chimpanzees were trained to touch the flashing part, and the other parts subsequently turned gray. (**e**) At the same time, another image of a chimpanzee body appeared in another location on the screen. Here, a different chimpanzee picture is used as an example, although the second pictures were sometimes the same as the first. (**f**) Chimpanzees touched one part of the second picture, and the other body parts turned gray. This example depicts them touching the arm area. This screen remained visible for a short period of time before all images disappeared and the starting key appeared again for the next trial.

To ensure that the chimpanzees would be able to select specific body parts, we used images of chimpanzee bodies in which all the major body parts were at a slight distance from each other (Figs. 1b and 4). All pictures were 360 px × 360 px, and the distance between each body part was approximately 10 px. The images were selected from a library of pictures that we had previously used to test the body inversion effect [Gao & Tomonaga, unpublished data], and they induced the inversion effect similar to typical body pictures. On this basis, we were confident of their validity as samples of chimpanzee bodies.

During the pre-training phase, we used three pictures in total. In the training phase, we used these three pictures in the first stage, three other pictures in the second stage, and a further three pictures in the third stage, for a total of nine pictures. These three picture sets depicted three chimpanzees in sitting postures, three in bipedal standing postures, and three in quadrupedal standing postures, respectively. In the testing phase, we used these nine pictures in the “familiar” trials, in which the sample and choice stimuli were exactly the same. In the “testing” trials, we used 10 pairs of novel pictures, 5 of which were same-picture pairs; the other five were different-picture pairs. One pair appeared in each testing trial, serving as the “sample” and “choice” chimpanzee bodies, respectively. Pictures from different pairs did not appear in the same trial. Among the five different-picture pairs, two had chimpanzees in similar body postures, two had chimpanzees in distinctive postures, and one had two quadrupedally standing chimpanzees facing different directions.

The original pictures were obtained from Kumamoto Sanctuary, Wildlife Research Center of Kyoto University. They were edited to produce the stimuli using Adobe Photoshop software (Adobe Systems, San Jose, CA, USA) and Pixelmator (Pixelmator Team Ltd., Vilnius, Lithuania). The images were rendered in black and white, and the luminance was adjusted to ensure that it was at a consistently similar level. The 16 different stimuli location patterns were counterbalanced during the experiment.

### Procedure

We defined four major body parts: head, torso, both arms, and both legs. One of these four parts was exchanged quickly between the original color and yellow-hued colors to create a flashing visual effect to attract the chimpanzees’ attention; these served as an index for the sample part of each trial. The computer program recorded the chimpanzees’ touches on the screens and determined whether they corresponded to one of the body parts or fell outside. The areas of interest (body parts) were marked with invisible pentagons on each picture. The pentagons for each body part were placed slightly apart from each other to prevent accidental touch errors (Fig. 1b).

In the pre-training stage, we trained the chimpanzees to touch the flashing body parts. One training session consisted of 48 trials, with the flashing parts counterbalanced. In each trial, after touching a circle in the middle bottom of the screen (i.e., the start key), one chimpanzee body appeared with one body part (head, torso, both arms, or both legs) flashing three times. If the chimpanzees touched the flashing part, they received a food reward accompanied by a chime sound; the other parts turned gray while the touched part remained unchanged, thereby creating visual feedback for touches. This picture remained for 1.5 s and then disappeared. The inter-trial interval was 1.5 s. The next trial started with the appearance of the start key. If the chimpanzees touched a place that was not the flashing part, it flashed again until they made the correct choice. We recorded the touch accuracy of their first try. Each participant completed at least 15 sessions regardless of their performance. If they attained an accuracy of 37.5% (significantly greater than the chance level) for two consecutive sessions, they could proceed to the next phase. One chimpanzee, Chloe, was unable to satisfy the criterion after 30 training sessions, so we applied a correction from the 31st session—that is, we added one trial after a trial in which she failed to touch correctly on her first attempt. After her performance reached the criterion, we omitted the correction procedures to assess her performance. She completed three sessions with the correction procedure and satisfied the criterion in the next two sessions without correction procedures.

In the training stage, we trained the chimpanzees so that they were familiar with this body part matching-to-sample paradigm. One training session consisted of 48 trials with stimulus pictures, their locations, and target body parts being counterbalanced. In each trial, after touching the start key, one chimpanzee picture appeared, and one body part flashed. The chimpanzees were required to touch the flashing part. If they touched other parts, the body part flashed again until they touched it. Subsequently, all other parts turned gray, but that body part remained unchanged. Then, another picture, which was exactly the same picture as that which had appeared at the beginning of the trial, appeared in another location. The chimpanzees were required to touch one body part on this new picture. If they touched other parts, the program did not show any response. After they touched one body part, other parts turned gray, and the picture remained on the screen for either 1.5 s (correct choices, feeder working duration) or 2 s (incorrect choices, timeout period) as visual feedback, before both chimpanzee pictures disappeared from the screen and the next trial started. If the participants made a correct choice—that is, the part was the same as the flashing part on the chimpanzee body shown in the first picture—they immediately received a food reward accompanied by a chime sound. Otherwise, they received no food reward, and a buzzer sounded with a timeout of 2 s. The inter-trial interval was 1.5 s. If they had an overall accuracy of ≥ 85% and accuracies of ≥ 75% for each body part, for two consecutive sessions, they could proceed to the next phase where new pictures were used. If they reached the same criterion in this phase, they could proceed to the third phase, in which new pictures were used. After they completed all three phases, they completed confirmation sessions. Each confirmation session consisted of 36 trials (9 pictures × 4 body parts). The criterion to pass this was the same as above. Then, they could proceed to the next stage. Because Ayumu did not come to the lab often, he could not finish the training stage. The remaining five chimpanzees underwent the tests in the final stage.

In the final stage, testing, we combined trained and novel trials in each session (Fig. 4). Each session included 36 trained trials from the training stage (9 pictures × 4 body parts) and 8 test trials (2 pairs × 4 body parts). The two pairs in the test trials contained one pair of the same picture and one pair of different pictures. Each test trial was followed by trained trials. The locations of the test trials in the session sequence were not fixed, but there were at least two trained trials, and at most six trained trials, between every two test trials. In total, each chimpanzee completed 60 sessions, which provided 12 repetitions for each body part in each picture pair. They completed three sessions on each testing day, and no picture pairs were repeated within a day.

### Data analyses

When analyzing the data from the testing stage, we first checked how accurately the participants could match the body parts.

We compared the participants’ performances with the chance level via binomial tests for each individual and condition (“baseline” [trained pairs], “test_same” [novel pictures but each pair had the same picture], and “test_different” [novel pictures and each pair had two different pictures]). We then applied this analysis to the data from the “test_different” condition and checked each individual’s results for each body part. Because the stimuli were repeated several times, we also examined the chimpanzees’ performance in their first encounters with each pair using binomial tests.

Next, we mixed all the data together and performed generalized linear mixed model (GLMM) tests using R (R Core Team, Vienna, Austria) and the “lme4” package^31,32^ to examine the participants’ accuracy and response times, respectively. The accuracy data had a binomial distribution, and the response time data had a gamma distribution. We set condition, body part, and their interaction as the fixed effects and participant ID, session number, trial number, sample picture, and picture location as random effects in the full models. The null models only included the above random effects; there were no fixed effects. We compared the full model and the null model first. If they differed significantly, we examined the significance of each fixed effect. Because there were significant interactions between condition and body part for both the accuracy and response time data, we then conducted pairwise comparisons of all relevant pairs to further examine the situation ([6 comparisons (for the 4 body parts)] × 3 conditions + [3 comparisons (for the 3 conditions)] × 4 body parts = 30 pairs in total).

To visually represent the chimpanzees’ discrimination and body part categorization performance, we performed a multi-dimensional scaling analysis of all individuals’ data in the “test_different” condition using SPSS for Windows (version 28.0; IBM Corp., Armonk, NY, USA). The “choice distances” were calculated from the choices made for each body part type.

### Supplementary Information

Supplementary Information 1.

Supplementary Information 2.

## Data Availability

Raw data is available in the supplementary materials.
