# Supplementary material for: Body part categorical matching in chimpanzees (Pan troglodytes)
Source: Sci Rep. 2024 Jul 10;14:15896. doi: 10.1038/s41598-024-66829-w (PMC11236962; doi:10.1038/s41598-024-66829-w)
Supplement: Supplementary file 2 — Supplementary Information 2. [file 41598_2024_66829_MOESM2_ESM.pdf]

Body part categorization in chimpanzees (*Pan troglodytes*)

Supplementary material: Stimuli

Jie Gao, Ikuma Adachi

Kyoto University

In this supplementary material, we present the stimuli used in this study. We also present summary figures of accuracy and response time (of correct trials) for each pair of the "novel" stimuli in the testing stage.

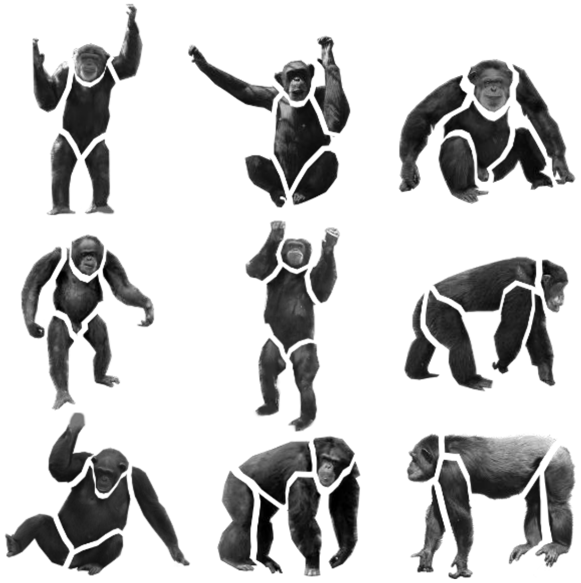

Fig. S1 The stimuli used in the training stage

From the top row to the bottom row, every three stimuli were used in each phase of training. They were then used again in the next stage as “baseline” stimuli. (Credit of the photos that these stimuli were modified from: Kumamoto Sanctuary, Wildlife Research Center, Kyoto University.)

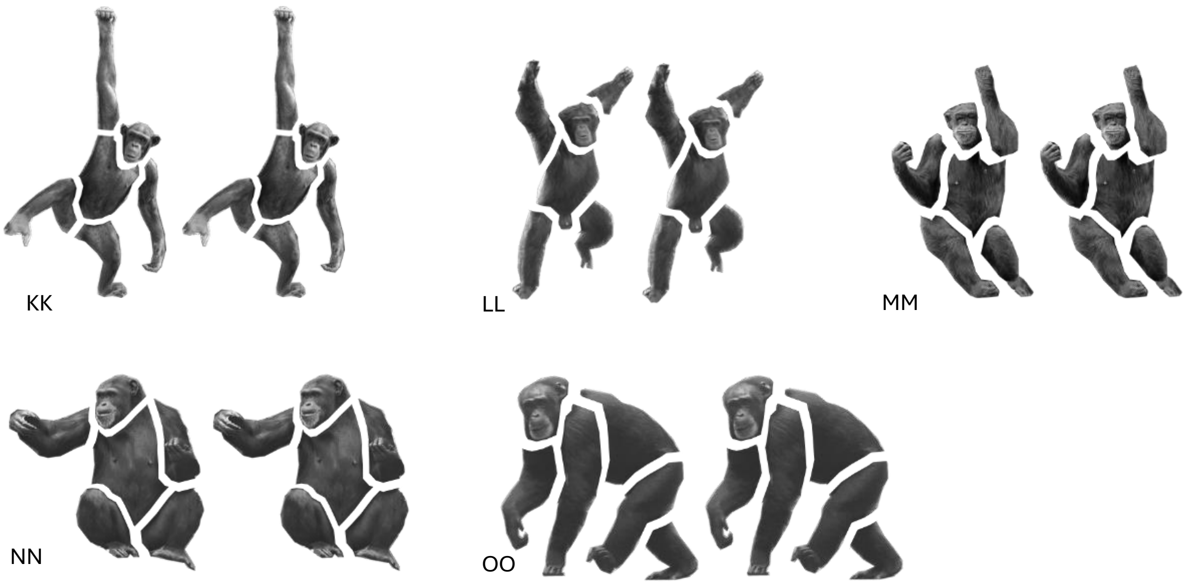

Fig. S2 The “test_same” stimuli

This figure shows the stimuli used in the “test_different” trials in the testing stage. The letters represent the name of the stimuli. Each cluster represents one fixed pair used in the experiment. For example, picture KK always appeared with picture KK during one trial. (Credit of the photos that these stimuli were modified from: Kumamoto Sanctuary, Wildlife Research Center, Kyoto University.)

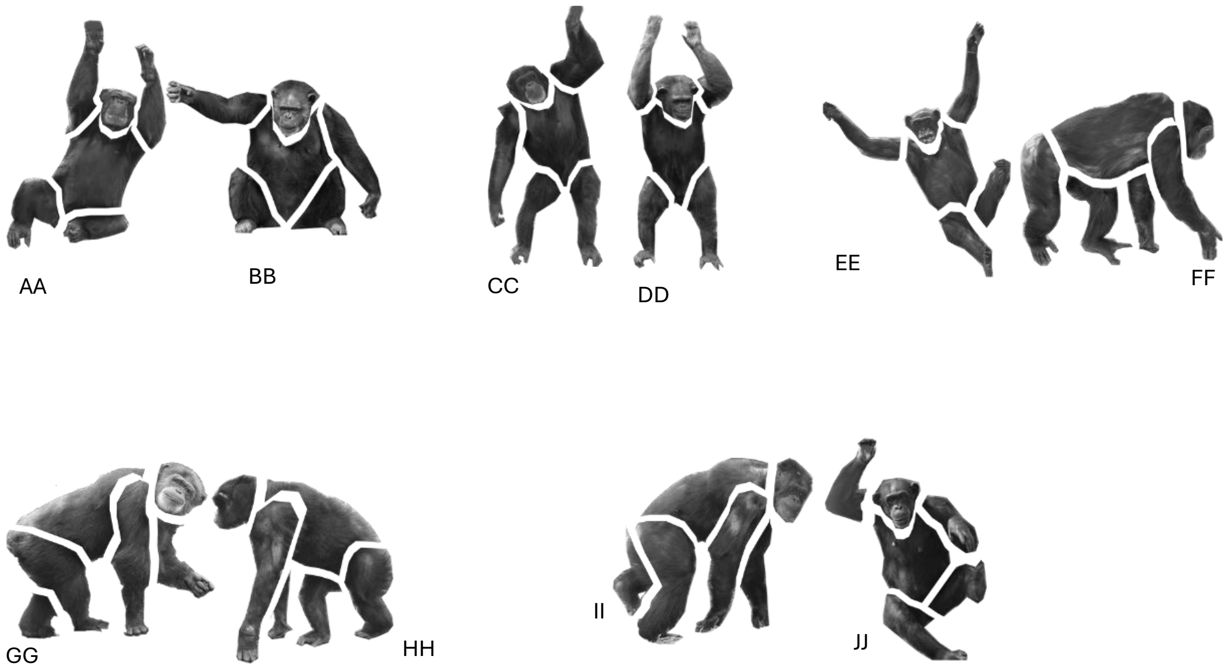

Fig. S3 The “test_different” stimuli

This figure shows the stimuli used in the “test_different” trials in the testing stage. The letters represent the name of the stimuli. Each cluster represents one fixed pair used in the experiment. The pairs were: AA-BB, CC-DD, EE-FF, GG-HH, and II-JJ. (Credit of the photos that these stimuli were modified from: Kumamoto Sanctuary, Wildlife Research Center, Kyoto University.)

Table S1 The correct trials of each stimulus pair of each chimpanzee (total trial number: 60) and the results of binomial tests compared with the chance level (25% correct)

Pair \ Chimpanzee		Chloe	Cleo	Pal	Ai	Pen
AA-BB	Correct trials	42	46	40	35	41
	P	< .001	< .001	< .001	< .001	< .001
CC-DD	Correct trials	40	38	40	41	43
	P	< .001	< .001	< .001	< .001	< .001
EE-FF	Correct trials	30	35	25	35	34
	P	< .001	< .001	0.001	< .001	< .001
GG-HH	Correct trials	41	36	33	34	33
	P	< .001	< .001	< .001	< .001	< .001
II-JJ	Correct trials	29	35	30	26	29
	P	< .001	< .001	< .001	< .001	< .001
KK-KK	Correct trials	46	44	38	44	44
	P	< .001	< .001	< .001	< .001	< .001
LL-LL	Correct trials	45	41	45	45	44
	P	< .001	< .001	< .001	< .001	< .001
MM-MM	Correct trials	45	43	45	43	44
	P	< .001	< .001	< .001	< .001	< .001
NN-NN	Correct trials	47	49	47	41	47
	P	< .001	< .001	< .001	< .001	< .001
OO-OO	Correct trials	39	43	43	39	40
	P	< .001	< .001	< .001	< .001	< .001

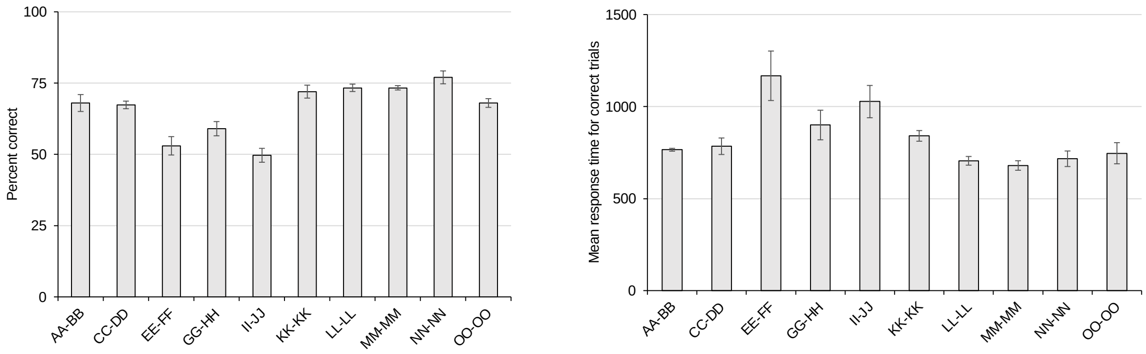

Fig. S4 Accuracy and mean response time data for each "novel" pair

Data summarized by each individual for each pair of stimuli. Error bar: SEM.

From Table S1 and Fig. S4, it is clear that for each stimulus pair, the chimpanzees' performances were above the chance level. There is also a tendency of a worse performance in the pairs EE-FF and II-JJ, which probably reflects the difficulties brought by different postures of the two images in these pairs.
